# Management of imported malaria in Europe

**DOI:** 10.1186/1475-2875-11-328

**Published:** 2012-09-17

**Authors:** Helena H Askling, Fabrice Bruneel, Gerd Burchard, Francesco Castelli, Peter L Chiodini, Martin P Grobusch, Rogelio Lopez-Vélez, Margaret Paul, Eskild Petersen, Corneliu Popescu, Michael Ramharter, Patricia Schlagenhauf

**Affiliations:** 1 Department of Medicine Solna/Unit for Infectious Diseases, Karolinska Institutet, Stockholm, Sweden; 2 Department of Communicable Disease Control and Prevention, Stockholm County Council, Stockholm, Sweden; 3 Intensive Care Unit, Centre Hospitalier de Versailles, Site André Mignot, 177 rue de Versailles, Le Chesnay 78150, France; 4 Bernhard-Nocht-Institut für Tropenmedizin, Hamburg, Germany; 5 University Division of Infectious and Tropical Diseases, University of Brescia and Spedali Civili General Hospital, Brescia, Italy; 6 Hospital for Tropical Diseases and London School of Hygiene and Tropical Medicine, London, UK; 7 Center for Tropical Medicine and Travel Medicine, Department of Infectious Diseases, Academic Medical Center, University of Amsterdam, Amsterdam, The Netherlands; 8 Tropical Medicine & Clinical Parasitology. Infectious Diseases Department.Hospital Ramón y Cajal, Madrid, Spain; 9 Department and Clinic of Tropical and Parasitic Diseases, University of Medical Sciences, Poznan, Poland; 10 Department of Infectious Diseases, Aarhus University Hospital Skejby, Aarhus, Denmark; 11 Clinical Hospital of Infectious and Tropical Diseases "Dr.Victor Babes", University of Medicine and Pharmacy "Carol Davila" Bucharest, Bucharest, Romania; 12 Department. of Medicine I, Div. of Infectious Diseases and Tropical Medicine, Medical University of Vienna, Vienna, Austria; 13 University of Zürich, Centre for Travel Medicine, Division of Epidemiology and Communicable Diseases, Zürich, Switzerland

## Abstract

In this position paper, the European Society for Clinical Microbiology and Infectious Diseases, Study Group on Clinical Parasitology, summarizes main issues regarding the management of imported malaria cases. Malaria is a rare diagnosis in Europe, but it is a medical emergency. A travel history is the key to suspecting malaria and is mandatory in patients with fever. There are no specific clinical signs or symptoms of malaria although fever is seen in almost all non-immune patients. Migrants from malaria endemic areas may have few symptoms.

Malaria diagnostics should be performed immediately on suspicion of malaria and the gold- standard is microscopy of Giemsa-stained thick and thin blood films. A Rapid Diagnostic Test (RDT) may be used as an initial screening tool, but does not replace urgent microscopy which should be done in parallel. Delays in microscopy, however, should not lead to delayed initiation of appropriate treatment. Patients diagnosed with malaria should usually be hospitalized. If outpatient management is preferred, as is the practice in some European centres, patients must usually be followed closely (at least daily) until clinical and parasitological cure. Treatment of uncomplicated *Plasmodium falciparum* malaria is either with oral artemisinin combination therapy (ACT) or with the combination atovaquone/proguanil. Two forms of ACT are available in Europe: artemether/lumefantrine and dihydroartemisinin/piperaquine. ACT is also effective against *Plasmodium vivax*, *Plasmodium ovale*, *Plasmodium malariae* and *Plasmodium knowlesi*, but these species can be treated with chloroquine. Treatment of persistent liver forms in *P. vivax* and *P. ovale* with primaquine is indicated after excluding glucose 6 phosphate dehydrogenase deficiency. There are modified schedules and drug options for the treatment of malaria in special patient groups, such as children and pregnant women. The potential for drug interactions and the role of food in the absorption of anti-malarials are important considerations in the choice of treatment.

Complicated malaria is treated with intravenous artesunate resulting in a much more rapid decrease in parasite density compared to quinine. Patients treated with intravenous artesunate should be closely monitored for haemolysis for four weeks after treatment. There is a concern in some countries about the lack of artesunate produced according to Good Manufacturing Practice (GMP).

## Background

Malaria continues to pose challenges in diagnosis and management and remains an infrequently encountered infection for many physicians in non-endemic areas. Malaria symptoms are non-specific and cannot easily be distinguished from a wide range of other febrile conditions on clinical grounds alone [1]. Thus, a high degree of suspicion is needed and a travel history is essential in any febrile patient presenting with non-specific, often flu-like symptoms and signs.

Malaria cannot be confirmed clinically. The gold standard for malaria diagnosis is light microscopy of Giemsa-stained thin and thick blood films, which requires a high level of expertise. This is not always available around the clock outside specialized centres and rapid diagnostic tests (RDTs) are, therefore, increasingly used. However, RDTs have limitations and once a malaria diagnosis has been made, the infecting species present must be determined. Furthermore, in the case of *Plasmodium falciparum* or *Plasmodium knowlesi*, the parasitaemia (expressed as number of parasites per microlitre of blood or as a percentage of red cells infected) is an essential parameter for determining whether the patient has complicated malaria or not, and to monitor treatment effect.

The management of malaria in non-endemic areas may vary between centres. For example, in a retrospective analysis of over 500 patients from five European countries treated between 2003 and 2009, 18 different combination regimens were used [2]. To standardize management based on current evidence, this paper reviews malaria management for non-specialists and advocates how it should be practised in Europe.

### Short epidemiology of imported malaria

Malaria is one of the major global public health challenges with an estimated 225 million clinical cases and more than 655.000 deaths in 2011, mainly in children aged less than five years old from sub-Saharan Africa. However, recent studies have found that the mortality may be grossly underestimated [3-5]. In Europe, malaria has been eradicated except in Azerbaijan, Georgia, Kyrgyzstan, Tajikistan and Turkey [6]. It is estimated that 25–30 million individuals travel annually from Europe to areas with malaria transmission.

Malaria imported to Europe is seen in travellers returning from or migrants coming from endemic areas and migrants living in Europe returning from visiting friends and relatives (VFR). VFR children are particularly at risk. In 2010, WHO reports that 6,244 cases of malaria were imported in Europe down from 14,703 cases in 2000 [6], however, this is considered to be an underestimation and the numbers may be six-fold greater [3] and this decrease in numbers contradicts national studies, which all indicate an increasing number of imported cases. Latest data from the UK [7] and Europe [8] show increasing trends in imported malaria and the latest US statistics show an increase of 14% in imported malaria for 2010 [9]. The majority of imported cases remains uncomplicated [1], and the mortality of imported *Plasmodium falciparum* malaria cases varies from 0.4% in a large cohort from France up to 5% in a recent cluster of cases imported from The Gambia [10,11].

The malaria programme in the WHO European Region, reported an eight-fold increase in the number of imported malaria cases between 1972 and 1988 (from 1,500 to 12,000 cases), followed by a more gradual rise in 2000 (15,500 cases) with France, the UK, Germany and Italy accounting for more than 70% of all cases [12]. One study found that the crude risk for travellers varied from 1 per 100,000 travellers to Central America and the Caribbean to 357 per 100,000 in Central Africa [13].

More than five million African migrants may currently be living in Europe, one third of them originating from sub-Saharan Africa [14]. The proportion of imported malaria cases due to migrants in Europe has increased during recent years from 14% to 83% [15-18]. VFRs travelling to sub-Saharan Africa have more than eight times the risk of being diagnosed with malaria compared to tourists, and more than twice the odds of being diagnosed of malaria after travel to Asia [19]. VFR children are particularly at risk [20].

Malaria infection among migrants, for example, asymptomatic individuals with sub-microscopic parasitaemia, could increase the risk of transmission leading to re-introduction of malaria in certain areas that have competent vectors and climatic conditions as seen in Greece in 2011 [21]. Moreover, imported malaria infections in migrants can also play a role in non-vectorial transmission, through blood transfusion, organ transplantation or occupational exposure.

Migrants commonly believe they are immune to malaria [22], but their immunity wanes after arriving in Europe. The low risk perception means that this group rarely seeks pre-travel advice [7,23-25]. The incidence of *P. ovale* infections and mixed infections is very similar to the incidence found in West Africans [26-29].

Rare modes of transmission mean that patients with fever and without a travel history to endemic areas might need to be tested for malaria. These include so called “airport malaria” where *Anopheles* mosquitoes carrying malaria parasites are transported by aeroplane to a non-endemic area and take a blood meal from someone living close to the destination airport [30,31], and “baggage malaria” [32]. Malaria parasites can also be transmitted in blood as a consequence of intravenous drug use [33]. Transmission of malaria by blood transfusion from asymptomatic carriers is a major problem in tropical Africa [34] and febrile patients with a history of blood transfusion from a donor in or from a malaria-endemic area should be suspected to have malaria until proven otherwise. Congenital transmission or transmission by organ transplantation may also occur [35].

### Pathophysiology

The pathophysiology of malaria involves the cytoadherence of infected erythrocytes (discussed below) and consequent microvascular obstruction, as well as destruction of infected erythrocytes and the host’s response to the released substances. Chills and fever are associated with high levels of tumour necrosis factor released from monocytes stimulated by antigens released by bursting schizonts. Thrombocytopenia may be immune-mediated or due to activation of the coagulation cascade [36]. Anaemia is a consequence of intravascular lysis of infected erythrocytes, impaired bone marrow responses and increased clearance of uninfected red cells.

*P. falciparum*-infected erythrocytes adhere to microvascular endothelium in a process known as sequestration, which allows them to avoid being removed in the spleen. This is favoured by other processes, such as a reduction of red cell deformability, rosetting of non-infected erythrocytes around the infected erythrocytes, platelet-mediated aggregation of infected erythrocytes [37-41]. Sequestration is seen especially in the small venules of vital organs (brain - predominantly in white matter, heart, lung, liver, kidneys, eyes) [42,43]. Sequestering of the erythrocytes in areas with low oxygen tension may favour survival of the parasites. It has been proposed that cerebral malaria is part of a heterogeneous clinical presentation involving multi-system dysfunction and acidosis [44]. A study of volume depletion in children with malaria demonstrated that lactate (arterial) was increased at admission [45], that volume depletion was not severe and that lactate is the best indicator for tissue perfusion and acidosis.

### Clinical symptoms

#### Clinical symptoms in non-immunes (persons not born and raised in endemic areas)

Most infections due to *P. falciparum* become symptomatic within 30 days after return from the malaria endemic area, but longer incubation periods are seen with the other species and are prolonged by incomplete malaria chemoprophylaxis which may suppress parasitaemia without achieving full protection. A recent study from Portugal including 284 patients (46% non-immunes and 54% semi-immunes) found that the diagnosis was made between the day of return from the malarious area and up to 47 days later; a single non-immune patient was first diagnosed on the 120th day after leaving Angola [46].

Prodromal symptoms, which may precede the fever for up to two days are fatigue, loss of appetite, headache and body pains. In non-immune patients, malaria usually starts suddenly with a severe feeling of sickness and fever - often reaching 39°C and higher [1]. Not all patients show typical fever paroxysms and absence of fever does not remove the suspicion of malaria in an ill patient. A regular fever pattern is not always present. If present, the frequency of the febrile episodes depends on the parasite species, occurring every 48 hours (tertian) for *P. vivax* and *P. ovale*, every 72 hours (quartan) for *P. malariae* and 24 hours (quotidian) for *P. knowlesi*. In *P. falciparum* malaria the fever usually lacks a regular pattern.

Common symptoms are headache and myalgia. Other symptoms may include nausea, vomiting, dry cough, icterus, confusion and respiratory distress. Compromised circulation leads to renal failure and impaired tissue perfusion resulting in acidosis. Gastrointestinal complaints unrelated to treatment, including vomiting and diarrhoea are less frequent. Patients with significant fever paroxysms may initially have a normal temperature between the fevers and feel relatively well.

Clinical examination is non-specific or even completely unremarkable. It often takes some days before anaemia or hepatosplenomegaly develop. Coryza, swelling of lymph nodes and eosinophilia are not seen in malaria.

#### Clinical symptoms in persons migrating from malaria endemic areas

Malaria in adult migrants is characterized by a milder clinical presentation, lower levels of parasitaemia, shorter parasite clearance time after treatment and shorter fever duration compared to malaria in travellers due to previously acquired semi-immunity [29,47-49]. Children born in Europe to migrant parents are not immune.

A high proportion of migrants have few symptoms and present long after arrival in the host country [50], with periods of months up to more than 14 years recorded [50-54]. If semi-immunity is lost, migrants who travel to their country of origin would have a risk of clinical malaria approaching that of travellers born in non-endemic countries, however a degree of clinical immunity against severe malaria is often retained. A high prevalence (from 7.1% to 31.8%) of *P. falciparum* infection (detected by PCR) has been found among asymptomatic sub-Saharan African migrants after their migration to Europe [48,55,56].

#### Indications for malaria diagnosis

Diagnostic tests for malaria should be performed in any ill patient who has a history of exposure, i.e. patients with a history of travel to malaria-endemic areas, whether or not they are febrile at presentation [57].

Pregnant women visiting endemic areas or arriving from areas of malaria transmission are at greater risk of clinical malaria during pregnancy [50] than non-pregnant women. Malaria parasites cross the placenta and consequently the disease can occur in newborns from asymptomatic mothers [58,59]. Splenectomized patients may have more severe symptoms [60].

#### Laboratory results (other than tests for malaria parasites)

Most parameters are non-specific. Normochromic normocytic anaemia is observed in the majority of infected patients, but is rare in the first few days of illness. The most consistent finding is thrombocytopaenia and a study from the UK found that children with malaria and a platelet count of < 50,000 per ml had an odds ratio of 8.3 for admission to intensive care units [61]. As the infection progresses, haemolysis is indicated by elevated LDH, free haemoglobin and low haptoglobin.

Although commonly unaffected except in case of a secondary infection (often with non-typhoidal salmonellae), the leucocyte count may be raised or greatly diminished in very severe malaria cases and there may be slight monocytosis and lymphocytopenia. The C-reactive protein, procalcitonin, fibrinogen, orosomucoid and cytokine levels are raised in acute malaria. Thrombocytopaenia with a lower concentration of fibrinogen and elevated fibrin degradation products strongly suggest disseminated intravascular coagulation (DIC). Moderate hyponatraemia can be seen, but plasma potassium level is normal [62]. Bicarbonate concentration is reduced and lactate may be elevated in metabolic acidosis, and arterial lactate is a better marker for acidosis than venous standard bicarbonate. Serum creatinine and urea, total and conjugated bilirubin and liver transaminases may be raised, and a slight elevation of hepatic alkaline phosphatase may be seen. Hypoglycaemia may occur and, in the absence of quinine treatment is accompanied by other signs related to high parasitaemia and severe acidosis [63]. Laboratory indicators of a poor clinical prognosis in severe malaria cases are shown in Table 1.

**Table 1:** Laboratory indicators of a poor clinical prognosis in severe malaria


Hyperparasitaemia:	> 250.000/ul or > 2% of infected erythrocytes in non-immunes and > 5% in semi-immune individuals
	Schizonts of *P. falciparum* in peripheral blood
	Mature pigmented parasites (> 20% of parasites)
Peripheral blood leukocytes	with ingested hemozoin (>5%)
Haemoglobin	< 5 g/dl or packed cell volume < 0.15
Polymorphonuclear leukocytes	> 12.000/ul
Coagulation disturbancies	Platelets < 50.000/ul
	Prothrombin time prolonged > 3 sec
	Prolonged partial thromboplastin time
	Fibrinogen < 200 mg/dl
	Low antithrombin III levels
Hypoglycaemia	< 2.2 mmol/l (< 40 mg/dl)
Acid-base disturbancies	Venous HCO3 < 15 mmol/l and/or arterial pH < 7.3
	Lactate > 5 mmol/l
Kidney function	Serum creatinine > 3.0 mg/dl (> 265 mmol/l)
	Blood urea nitrogen > 60 mg/dl
Liver function	More than 3-fold elevation of aminotransferases (AST, ALT)
Cerebrospinal fluid	High CSF lactic acid (> 6 mmol/l) and low CSF glucose concentration

Blood cultures should be obtained on admission as malaria infection can be complicated by septicaemia [64,65]. All patients with malaria should have the following obtained at the time of diagnosis: haemoglobin, MCV, MCHC, differential leucocyte count, platelets, blood urea nitrogen or creatinine, alanine transferase, basic phosphatase and LDH. In complicated malaria this should be supplemented with tests for DIC and arterial blood for pH, lactate (arterial), blood gases and blood cultures. Additional potentially useful parameters include chest x-ray, urine culture and urine leukocytes, ECG, potassium, urea, ALT, LDH, haptoglobin, fibrinogen.

#### Diagnostic procedures for malaria parasites including parasitaemia

Microscopic examination of Giemsa-stained thin and thick blood films remains the gold standard because it is rapid, easy to perform and sensitive [66] with a sensitivity down to five parasites per microlitre in expert hands [67]. Thick blood films are combined with thin blood films as identification of the infecting species is much more easily accomplished using a well-stained thin film. However, the volume of blood examined in 100 high power fields on a thick blood film is approximately 50 times more than in 100 high power fields of a thin blood film, so malaria cannot be excluded based on negative examination of only a thin blood film.

Early diagnosis is important to prevent uncomplicated malaria progressing to complicated disease. Patients with malaria should be managed in centres with the ability to quantify the parasitaemia (usually expressed as the number of parasites per microlitre of blood or as the percentage of red cells containing malaria parasites). After the start of treatment, there is a lag-phase before the parasite density begins to decline [68] and there may even be an increase in the first 24 hours after starting treatment.

For falciparum malaria, many RDTs show 100% parasite detection score down to a parasite density level of 200 parasites per microlitre, equivalent to a parasitaemia of approximately 0.004% [69]. Polymerase chain reaction (PCR) can detect parasites down to a density of 0.01 parasites per microlitre after a lysis procedure and 1 parasite per microlitre without lysis [70]. However, PCR analysis is not instantly available around the clock so in practice, diagnosis relies on RDTs and light microscopy of Giemsa-stained thin and thick blood films. PCR is, however, very useful in partially treated cases, in sub-microscopic malaria in immigrants and in detection of mixed parasitaemia.

RDTs are increasingly used in medical centres with limited access to experienced microscopists, however, a rapid test cannot determine the parasite density. False negative RDTs in patients with very high parasite densities have been described, probably due to the so-called “pro-zone” phenomenon [71,72]. This problem seems to be limited to tests based on detection of Histidine Rich Protein 2, HRP2 [71]. Mutations in the HRP2 gene may also result in false negative tests [73,74] and rheumatoid factor may lead to false positives [75]. Assays are available that detect all species i.e. *P. falciparum, P. vivax, P. ovale, P. malaria*e, and *P. knowlesi* based on the detection of pan-malarial antigens aldolase and LDH antigen [76]. *Plasmodium knowlesi* infections will be detected by rapid tests which include the pan plasmodial aldolase or LDH antigens [69,77]. The latest results of the WHO multi-centre evaluation of different rapid diagnostic tests show that the best performance was found with tests based on a combination of the HRP2 and pan plasmodial proteins [69]. Clinicians using rapid tests should be instructed that no RDT test so far is 100% reliable and that they should be used in parallel to and not instead of blood film examination. In order to reduce the risk of missing malaria, testing with blood films and RDTs should be performed on three blood samples taken at daily intervals for patients with high suspicion for malaria. If the suspicion of malaria remains after three negative samples, expert advice should be obtained from a tropical or infectious diseases specialist. Once the diagnosis has been made, the patient should have daily blood films until they are negative for asexual parasites (ie. rings, trophozoites, schizonts). Gametocytes do not multiply or cause clinical illness and may remain after clearance of the asexual parasitaemia.

#### Treatment of uncomplicated *P. falciparum* malaria

Treatment should provide rapid clinical and parasitological cure within three days. Oral ACT is the standard treatment of uncomplicated malaria as recommended by WHO [78,79] (Table 2). Currently, artemether/lumefantrine, and dihydroartemisinin/piperaquine, a ACT formulation registered by the European Medicines Agency, EMA, are licensed for use in Europe. Artemether/lumefantrine is the most widely-used ACT globally, is well tolerated and highly efficacious in all endemic regions except for *P. falciparum* infections acquired in Cambodia and the border regions of Thailand with Myanmar, where multi-drug resistant *P. falciparum* strains are highly prevalent.

**Table 2:** **Treatment of uncomplicated falciparum malaria in adults**[79]

	**Adult Patients**		
	**Drug**	**Dosage**	**Comment**
**First line**	Artemether/Lumefantrine (Riamet™)	Twice daily for three days >35 kg: 4 tablets each 20 mg/120 mg for 6 doses (0–8–24–36–48–60 hours)	Take with fatty food, reduced efficacy in Cambodia and border regions of Thailand
	Dihydroartemisinin/Piperaquine (Eurartesim™)°	Once daily for three days 36 <75 kg: 3 tablets each 320 mg/40 mg, 75-100 kg: 4 tablets each 320 mg/40 mg, daily for three days	Administration without food, at least 3 hours from any meal
	Atovaquone/Proguanil (Malarone™)	Once daily for 3 days >40 kg: 4 tablets each 250/100 mg	Administration with fatty food
**Second line**	Quinine*/Doxycycline	Thrice daily 10 mg/kg quinine plus daily 200 mg doxycycline for 7 days	Loose drug combination, off-label use
	Quinine*/Clindamycin	Thrice daily 10 mg/kg quinine plus twice daily 10 mg/kg clindamycin for 7 days	Loose drug combination, off-label use
	Mefloquine (Lariam™)	Split total dose in 2–3 doses 6–8 hours apart 45-60 kg: 5 tablets (3 + 2 tablets) >60 kg: 6 tablets (3 + 2 + 1 tablets)	Administration after food intake monotherapy, which is not suitable for regions with multidrug resistant falciparum malaria (SE-Asia)

*Quinine dose provided as quinine sulphate °Eurartesim tablet strengths are Dihydroartemisinin/piperaquine 20 mg/160 mg and Dihydroartemisinin/piperaquine 40 mg/320 mg.

Artemether/lumefantrine has to be administered with fatty food to obtain optimal plasma drug concentrations [80]. Dihydroartemisinin/piperaquine is a newly registered ACT with proven high efficacy and a favourable tolerability profile. It has been extensively used in malaria-endemic regions and marketing in European countries has started. Dihydroartemisinin/piperaquine should be taken fasting (Table 3).

**Table 3:** Important food – anti-malarial drug interactions

**Drug**	**Dietary recommendations**	**Mechanism of interaction**	**Practical consequence**
Atovaquone	Take with fatty meal	Increased solubility and absorbtion	Increased drug levels
Artemether/lumefantrine	Take with food	Lumefantrine is lipophilic (fat soluble)	Up to 16-fold increased drug levels
Dihydroartemisinin/piperaquine	Take on an empty stomach (fasting)	Food increases absorption of piperaquine	Reduces risk of QTc prolongation
Doxycycline	Without milk	Milk chelates tetracyclines and reduces absorption	Low drug concentrations and treatment failure
Mefloquine	With food	Increased solubility and absorbtion	Increased drug levels
Primaquine	With food	Increased solubility and absorbtion	Increased drug levels

Atovaquone-proguanil can be used as first-line treatment for uncomplicated malaria and needs to be administered with fatty food to increase bioavailability (Table 3). Atovaquone/proguanil is relatively slow acting with considerably longer parasite clearance times compared to ACT (Table 4). Atovaquone/proguanil is the preferred treatment option for uncomplicated falciparum malaria from regions with artemisinin resistance (Cambodia, Thailand border regions).

**Table 4:** **Severe manifestations of *P. falciparum *malaria in adults (WHO 2000, adapted WHO 2011)**[77,78]

**Prognostic ** **value**	***Clinical manifestations and laboratory findings***	**Frequency**
(?) no data	**Prostration**	**+++**
+	**Impaired consciousness** (score <11 on the Glasgow Coma Scale)	**++**
+++	**Acute respiratory distress**	**+**
++	**Multiple seizures**	**+**
+++	**Circulatory collapse** (systolic blood pressure <80 mm Hg with features of peripheral circulatory failure)	**+**
+++	**Pulmonary oedema** (radiological)	**+**
++	**Abnormal bleeding** (clinically defined)	**+**
+	**Jaundice** (clinically defined or serum bilirubin >50 mol/L	**+++**
+	**Macroscopic haemoglobinuria**	**+**
+	**Severe anaemia** (haemoglobin <5 g/dL or haematocrit <15%)	**+**
+++	**Hypoglycaemia** (blood glucose concentration <2.2 mmol/L)	**++**
+++	**Acidosis** (pH <7.35 or plasma bicarbonate <15 mmol/L)	**++**
+++	**High plasma lactate** (>5 mmol/L)	**++**
++	**High parasitemia** (especially 2% in non-immune patients and 5% in semi-immune patients)	**+**
++	**Acute renal failure** (serum creatinine > 265 μmol/L and 24-hour urine output <400 mL)	**+++**

Second-line anti-malarial treatments used when first-line anti-malarials are not available or excluded due to other reasons such as intolerance include mefloquine monotherapy for infections originating from regions without established mefloquine resistance (high prevalence of mefloquine resistance is common in Thailand, Myanmar and Cambodia). The use of mefloquine at treatment doses has been associated with a significantly higher incidence of serious neuropsychiatric adverse events [81]. Combinations of quinine with either doxycycline or clindamycin are also considered second line treatment options.

Quinine drug combinations have excellent efficacy, but tolerability is generally poor due to prolonged treatment courses and the occurrence of characteristic adverse effects (cinchonism) [82]. The WHO guidelines consider quinine plus tetracycline or clindamycine as alternative first-line treatments, but the study group is of the opinion that ACT should be the first line choice in Europe [79].

The use of chloroquine is not recommended for the treatment of *P. falciparum* malaria because of widespread resistance. However, chloroquine remains effective in Haiti, Dominican Republic, Middle East and Central America north of the Panama Canal, and may be considered as an alternative treatment if ACT cannot be used.

In the case of failed anti-malarial chemoprophylaxis, an anti-malarial drug different from the chemoprophylactic drug taken should be used for treatment. Finally, it is important to note that despite the fact that oral anti-malarials are recommended for the treatment of uncomplicated malaria, it is sometimes necessary for the responsible physician to use intravenous treatment with artesunate or quinine as recommended for the treatment of severe malaria. This decision may be taken based on evidence of important co-morbidities, intractable vomiting, or on clinical concern of the physician. The clinical criteria for severe malaria are shown in Table 4. Recently, the WHO defined a parasitaemia of 2% or more as *severe malaria* in non-immunes and 5% or more in patients from endemic areas [79].

Patients suffering from *P. falciparum* malaria should, in general, be admitted to hospital, since monitoring of prognostic parameters including parasitaemia, treatment adherence and if needed, transfer to intensive care units, would normally be instantly available. Repeated monitoring of blood pressure, urinary output and oxygen saturation may be indicated. However, management as outpatients may be considered in uncomplicated cases in some healthcare systems where daily follow up until clearance of parasitaemia and fever and monitoring of treatment adherence can be undertaken. Persons migrating from malaria endemic regions may fall into this category.

#### Treatment of complicated falciparum malaria

The clinical criteria for severe malaria are shown in Table 4. Severe malaria may also be caused by species other than *P. falciparum,* especially *P. knowlesi*. *Plasmodium vivax* can be severe in non-immunes [83]. The criteria for the definition of severe malaria were determined by studies carried out in endemic areas and their relevance to imported malaria in Europe remains controversial. However, these criteria may be adapted to the European context [84].

#### Choice of drugs

Severe imported *P. falciparum* malaria is an emergency which may become rapidly fatal [85]. Intravenous artesunate is the drug of choice [79] and a recent Cochrane review concluded that treatment with artesunate significantly reduced the risk of death both in adults (RR 0.61, 95% Confidence Interval (CI) 0.50 to 0.75; 1664 participants, five trials) and children (RR 0.76, 95% CI 0.65 to 0.90; 5765 participants, four trials) [86]. Intravenous artesunate must be started immediately after the confirmation of the diagnosis and the patients transferred to the ICU for management.

#### Intravenous artesunate (IVA)

IVA is superior to intravenous quinine (IVQ) in overall survival and safer and simpler to administer [86-89]. IVA contains artemisinin hemisuccinate 60 mg/ml and is reconstituted with 3 to 5 ml dextrose 5% and immediately administered in a bolus. IVA is administered as 2.4 mg per kilogram of body weight every 12 hours on day 1 and then once daily up to the total dose of 12 mg per kilogram administered in five doses over 3 days [80]. IVA should be the drug of choice for treatment of severe imported malaria in Europe even though trials have only been performed in endemic regions [89]. A recent study reported haemolytic anaemia in six out of 25 patients treated with IVA for severe imported malaria diagnosed 14–31 days after the first dose of IVA [90]. A larger study including 55 patients with severe malaria reported late onset haemolytic anaemia in six patients (9%) between 7 and 31 days after start of IVA [91] and three more cases have just been reported [92]. Nevertheless, in a large French study about 400 severe malaria patients treated with IVQ in the ICU, 28.5% of them required red blood cell transfusion for marked anaemia [84]. Until further data are available, patients should be monitored for four weeks following IVA for haemolysis and leukopaenia. IVA should be completed with a full course of ACT, atovaquone/proguanil or mefloquine.

IVA produced under European Good Manufacturing Practice (GMP) standards is not yet available. Nevertheless, in some European countries (particularly in France), IVA manufactured in China (Guilin Pharmaceuticals) has been approved by the National Drug Agency and is now available with a specific temporary authorization of use (see section: «unlicensed drugs » below).

#### Intravenous quinine (IVQ)

IVQ is the drug of choice if IVA is not immediately available. An ECG should be obtained before starting IVQ. Use of a loading dose is recommended to rapidly obtain a therapeutic serum quinine level [84]. The loading dose is 20 mg/kg quinine dihydrochloride in 10% glucose or 0.9% sodium chloride infused over 4 hours. Treatment is continued with 10 mg/kg quinine dihydrochloride by infusion over 4 h in 500 ml of 5% glucose, every 8 h until parasitaemia is less than 1% and the patient can take oral medication. Any previous treatment with mefloquine or quinine and/or an increased corrected QT interval >25%, are contra-indications for a loading dose because of an increased risk of cardiotoxicity. Continuous monitoring of the cardiac rhythm is necessary. The only strict contra-indications to IVQ are a documented previous history of blackwater fever [93], hypersensitivity to quinine, and cardiac arrythmia. Blackwater fever may occur on quinine treatment [93].

A full seven day course of IVQ is rarely completed. Quinine treatment can be changed either to an ACT or to an oral quinine-antibiotic combination as soon as the parasite density decreases and the patient tolerates oral treatment. The same principles apply for intravenous artesunate, where treatment is switched to oral ACT as soon as the parasite density has fallen adequately. In adults, doxycycline (or clindamycin during pregnancy) should be used in association with quinine if the quinine course cannot be followed by a course of ACT. Mefloquine should be avoided in patients with cerebral malaria even in the recovery phase. IVQ should be completed with a full course of ACT, atovaquone/proguanil or mefloquine.

#### Supportive care on ICU (Intensive Care Unit)

Amongst the various scoring systems for adult malaria patients requiring ICU treatment outside endemic areas, SAPS II and the WHO score appear to be most reliable [46,84,94]. Fluid management is very important. Fluid overload may cause pulmonary oedema [95]. The intravascular volume should be high enough to ensure sufficient systemic perfusion, but overhydration has to be avoided, and adults with severe malaria are very vulnerable to fluid overload [79]. Monitoring of plasma lactate is mandatory. For children, the FEAST trial provided high quality evidence that during paediatric malaria fluid bolus significantly increases mortality [96]. The maintenance of a particular central venous pressure in severe malaria cannot be supported by available studies [97]. In adults with severe *P. falciparum* malaria there was no observed improvement in patient outcome or acid–base status with fluid loading. Neither CVP (Central venous Pressure) nor PAoP **(**Pulmonary Artery occlusion Pressure**)** correlated with markers of end-organ perfusion or respiratory status [98]. In the shocked and/or acidotic patient with severe malaria, bacterial co-infection should be sought by blood culture and antibiotic treatment started urgently [94,99].

Acute renal impairment and failure is frequent and indications for acute dialysis do not differ from acute renal failure in other conditions. Hyponatraemia is often seen in falciparum malaria, caused by reduced kidney function and consecutive dilutional hyponatraemia or by an Anti Diuretic Hormone Syndrome in the case of euvolemia. In both cases, treatment is by fluid restriction. Precise monitoring of the fluid balance is essential.

In the case of cerebral malaria the usual supportive measures practiced in neurological intensive care medicine are recommended. However, corticosteroids as well as mannitol should *not* be given, as they lead to prolongation of coma time and worsen the prognosis [79]. Hypoglycaemia is often seen, especially with quinine therapy and the blood sugar has to be monitored closely. Mefloquine should be avoided in patients with cerebral malaria even in the recovery phase because of the risk of post-malaria neurological syndrome.

There is no consensus on the indications, benefits and dangers involved in exchange blood transfusion, so it should not be used [79]. Automated red blood cell exchange (i.e. erythrocytopheresis) is another potentially useful adjunctive treatment option to rapidly reduce high parasitaemia by removing infected erythrocytes. It has the advantage of less interference with volume and electrolyte status of the patient, but no randomized controlled trial has been conducted so far [100,101] and its role is unclear since the advent of ACT.

#### Unlicensed drugs

WHO guidelines recommend IVA in preference to quinine for the treatment of severe malaria in adults [80]. At present, no GMP (Good Manufacturing Practice) produced IVA is available in Europe. However, Guilin Pharmaceutical Factory No. 2 (Shanghai, People’s Republic of China), the manufacturer of the artesunate used in major trials in Southeast Asia and Africa [87,88], may supply the drugs upon request. Artesunate manufactured by Guilin has received pre-qualification from the WHO.

Since 2011, the French National Health Agency (AFSSAPS), now named (ANSM) has temporarily authorized the import and use of IVA (Malacef®) via ACR-Pharmaceuticals, the Netherlands, granting it a temporary authorization of use [102]. However, the use of non GMP artesunate remains sensitive from a legal point of view in many European countries, and some centres have addressed this by using a combination of quinine and artesunate, with satisfactory clinical outcomes and no safety concerns in a limited series of patients [103].

#### Paediatric malaria

Paediatric dosages are provided in Table 5. For imported cases, the risk of developing severe malaria is very high in VFR children without acquired semi-immunity and who are often more exposed to malaria [104]. Migrants’ children are less likely to complain of chills, arthralgia/ myalgia or headaches. The clinical approach to the treatment of children is comparable to adult patients and relies on the classification into uncomplicated and severe falciparum malaria (Table 2). The clinical assessment of young children may be more challenging in particular when assessing potential alteration of mental status. Prostration – the inability to walk, stand, sit, or feed – is a useful clinical indicator for severe disease in endemic regions, and it may be a particularly useful clinical indicator for very young children.

**Table 5:** Paediatric Patients

**Drug**	**Dosage**	**Comment**
Artemether/Lumefantrine (Riamet™, Riamet Dispersible™) (20/120 mg)	5–14 kg: 1 tablet per dose	Registered for treatment of patients of ≥5 kg body weight
	15–24 kg: 2 tablets per dose	Dispersible drug formulation is registered in Switzerland
	25–34 kg: 3 tablets per dose	
	> 35 kg: 4 tablets per dose	
	for 6 doses (0–8–24–36–48–60 h)^4^	
Dihydroartemisinin/Piperaquine (Eurartesim™)°	5 - < 7 kg: ½ tablet 160 mg/20 mg	Registered for treatment of patients of ≥5 kg body weight
	7 - < 13 kg 1 tablet 160 mg/20 mg	
		No paediatric drug formulation, but two strengths of tablets make use of this ACT feasible in children
	13 - < 24 kg: 1 tablet 320 mg/40 mg	
	24 - < 36 kg: 2 tablets 320 mg/20 mg	
	36 - < 75 kg: 3 tablets 320 mg/40 mg	
	75-100 kg: 4 tablets 320 mg/40 mg once daily for three days	
Atovaquone/Proguanil (Malarone™, Malarone Paediatric™)	5–8 kg: 2 tablets Malarone Paediatric	Registered for treatment of patients with >5 kg body weight
	9–10 kg: 3 tablets Malarone Paediatric	
	11–20 kg: 1 tablet Malarone	
	21–30 kg: 2 tablets Malarone	
	31–40 kg: 3 tablets Malarone	
	>40 kg KG: 4 tablets Malaroneonce daily for three days	
Quinine*/Doxycycline	Contraindicated in children below 8 years of age (13 years in some countries)	No drug registration
		Loose drug combination
Quinine*/Clindamycin	Thrice daily 10 mg/kg quinine plus twice daily 10 mg/kg clindamycin for 7 days	No drug registration
		Loose drug combination
Mefloquine (Lariam™)	5 - 10 kg: ½ - 1 tablet	Registered for treatment of patients of ≥5 kg body weight
	10–20 kg: 1–2 tablets	
	20–30 kg: 2–3 tablets (2 + 1)	
	30–45 kg: 3–4 tablets (2 + 2)	
	45–60 kg: 5 tablets (3 + 2)	
	> 60 kg 6 tablets (3 + 2 + 1)	
	20-25 mg/kg total dose divided in 1–3 doses 6 hours apart	

*Quinine dose provided as quinine sulphate.

°Eurartesim tablet strengths are Dihydroartemisinin/piperaquine 20 mg/160 mg (children) and Dihydroartemisinin/piperaquine 40 mg/320 mg (adults).

In line with recommendations for adults, ACT and atovaquone-proguanil are the recommended first-line treatments for uncomplicated *P. falciparum* malaria in paediatric patients in Europe (Table 5). Paediatric formulations should be used if available [105].

Quinine-clindamycin or mefloquine mono-therapy (except for regions with multi-drug resistant *P. falciparum* strains such as the Thai-Myanmar-Cambodia region) are appropriate second line drugs. Tetracyclines are contra-indicated in children below 8 years of age (below 13 years in some countries).

The administration of drugs in general and the intensely bitter taste of anti-malarials in particular (even more so if adult tablets are crushed to improve the ease of administration) is an important concern in young children. The use of paediatric drug formulations has been shown to improve the tolerability of antimalarials. To date, artemether/lumefantrine and dihydroartemisinin/piperaquine are useful first line paediatric anti-malarials. Artmether/lumefantrine is available as a dispersible tablet formulation (registered in Switzerland only). Atovaquone/proguanil is also available as paediatric tablets. Anti-malarial treatment of severe malaria in children follows similar algorithms as for adult patients and is based on prompt administration of intravenous artesunate (or quinine if artesunate is not available). Children presenting with malaria are likely to have high fever which increases the risk of vomiting and seizures. Fever should be reduced by tepid sponging and the use of an anti-pyretic such as paracetamol. which can be administered rectally, though paracetamol prolongs parasite clearance times in children.

Bacterial infections including sepsis and meningitis are more common in paediatric patients compared to adults in malaria endemic regions, and blood culture should be obtained on admission. Invasive non-typhoidal salmonella infections are among the most common invasive bacterial pathogens in some regions of Africa where malaria is highly endemic [106].

Congenital transmission of malaria to the newborn is a rare event and is estimated to occur in only 1% of newborns delivered by mothers with malaria. Clinical disease in the infant usually develops 2–8 weeks postpartum and includes non-specific symptoms such as fever, vomiting, diarrhoea, and poor feeding. To date, little information is available for the appropriate treatment of cases of congenital malaria and in patients of less than 5 kg bodyweight. Therefore, weight adjusted treatment with artemether/lumefantrine, dihydroartemisinin/piperaquine and atovaquone/proguanil may be considered for this indication.

#### Pregnant women

Pregnant women are at increased risk for malaria related morbidity and mortality. This increased risk extends to the post-partum period [107]. Pregnant women presenting with acute malaria require prompt and effective treatment. Malaria in pregnancy is no indication for caesarean section due to the low risk for vertical transmission. Suggested treatment for uncomplicated malaria in pregnant women is shown on Table 6. In complicated malaria, effective treatment with artesunate should be used to save the life of the mother even if there are safety concerns regarding the drug used. Quinine, chloroquine, clindamycin and proguanil are considered safe in the first trimester (Table 6). A recent database analysis of women exposed to mefloquine around the time of conception and in the first trimester showed no increased risk of malformations in offspring born to women who were exposed to mefloquine. Most of the women were exposed to chemoprophylactic doses rather than treatment doses of mefloquine [108].

**Table 6:** Malaria treatment in pregnancy

**Uncomplicated falciparum malaria**		
1^st^ trimester (1)	First line	Quinine-clindamycin quinine monotherapy
2^nd^ and 3^rd^ trimester	First line	artemether-lumefantrine
	Second line	quinine-clindamycin quinine monotherapy mefloquine (2)
**Complicated falciparum malaria**		
1^st^ trimester	First line	i.v. quinine (+ follow on treatment)
2^nd^ and 3^rd^ trimester	First line	i.v. artesunate (+ follow on treatment)
	Second line	i.v. quinine (+ follow on treatment)
***P. ovale, P. malariae*and*P. vivax*** (3)		
All trimesters	First line	Oral chloroquine
2^nd^ and 3^rd^ trimester	Second line	Oral ACT

1. Artemether/lumefantrine is not the first drug of choice due to lack of data on lumefantrine in pregnancy, but should be used if quinine is not available.

2. http://www.cdc.gov/malaria/new_info/2011/mefloquine_pregnancy.html.

3. Primaquine should not be used because of the risk of foetal haemolytic anaemia.

Pregnant women presenting with malaria in the first trimester should be treated with quinine and clindamycin for 7 days. Artesunate and clindamycin for seven days can be used if there is treatment failure with the quinine/clindamycin combination [80] (Table 6). Currently there is inadequate data on the use of ACT in the first trimester. Published data on 123 women exposed to ACT treatment in the first trimester showed no adverse outcomes. More data are available on ACT use in the second and third trimester (over 1,500 documented reports) and the WHO guidelines suggest that ACT should be used. Currently, there is insufficient evidence (154 cases) to recommend dihydroartesmisinin/piperaquine in pregnancy [109], so that artemether/lumefantrine is the only viable ACT option in Europe. Alternatives to artemether/lumefantrine in the second and third trimesters are quinine plus clindamycin or artesunate plus clindamycin [110] for 7 days in each case, or mefloquine monotherapy for regions without multidrug resistant parasites. Primaquine and tetracyclines should not be used in pregnancy. Atovaquone/proguanil is not recommended in pregnancy due to lack of data, but can be used in situations where no other drugs are available.

#### Treatment of *P. vivax*, *P. ovale*, *P. malariae* and *P. knowlesi*

*Plasmodium ovale* and *P. malariae* generally remain sensitive to chloroquine in all endemic areas, despite reports of delayed parasite clearance time [111]. *Plasmodium vivax* sensitivity to chloroquine has declined steadily in Indonesia, Peru and Oceania [79] and a paradigm shift is imminent, with opinion leaders beginning to call for a switch to ACT as the drug of choice in Indonesia, Peru and Oceania. The use of artemether/lumefantrine has been suggested as a pragmatic choice in areas with chloroquine-resistant *P. vivax*[112] and it may also be used in mixed infections of *P. falciparum* with this parasite or with *P. ovale* or *P. malariae*[113]. Mefloquine (15 mg/kg body weight as a single dose) has been found to be highly effective against *P.vivax* with a treatment success of 100% [114]. Monotherapy with doxycycline (100 mg twice a day for 7 days) results in poor cure rates against *P. vivax*[115]. Monotherapy with artemisinins alone should not be used, except for intravenous artesunate therapy in the initial stages of treatment for severe infection. Quinine is also effective against chloroquine resistant *P. vivax*, but it is not an ideal treatment because of low tolerability and it may lead to early relapses [114]. First-line treatment is chloroquine with ACT as second line if the response to chloroquine is poor.

*Plasmodium vivax* and *P. ovale* infections, but not *P. malariae*, require treatment with primaquine (PQ) for 14 days to eradicate liver hypnozoites and thus prevent relapses. *Plasmodium vivax* strains with reduced susceptibility to primaquine are found in southern regions of Oceania and South-East Asia and require a higher dose of primaquine (up to 0.75 mg/kg/day, max 30 mg per day) for 14 days to prevent relapses [116]. The CDC recommend routine use of 30 mg per day in adults tested negative for G6PD deficiency [117], and this should be standard treatment for adult patients with *P. vivax* and *P. ovale* after G6PD testing according to the CDC [117]. Other centres use the higher dose only for *P. vivax.* Primaquine should be administered concomitantly with the blood schizonticide or as soon as possible after treatment.

Primaquine is contraindicated in patients with deficiency of the enzyme glucose-6-phosphate dehydrogenase [118]. In patients with mild G6PD deficiency the WHO suggests using an intermittent primaquine regimen of 0.75 mg base/kg once a week for eight weeks [79,119]. Patients with significant G6PD deficiency should be referred for expert advice. A study of primaquine 0.5 mg/kg/day in children where G6PD deficiency was excluded found good tolerability [120].

Treatment of *P. knowlesi,* a well-know primate malaria species that has recently recognized as causing a significant number of human malaria cases in South-East Asia and especially Malaysian Borneo, is not standardized, and WHO has not yet provided recommendations [79]. Evidence is fast accumulating that, like *P.falciparum*, *P. knowlesi* may cause severe cases, with fatality rates as high as 27%, especially in older or female patients. Uncomplicated *P. knowlesi* cases can be treated with ACT, chloroquine, quinine, or atovaquone/proguanil [121]. Mefloquine may not be recommended in the light of case reports of treatment failure. There is no clear evidence of latent liver stages in *P. knowlesi*, and they have not been described in animal models [122,123]. A recent study showed that ACT cleared parasites faster than comparator antimalarials. In severe *P. knowlesi* cases, the use of IVA was associated with a lower case-fatality rate (17% vs 31%) and lower median parasite clearance time (2 days vs 4 days) than IVQ [124]. Thus uncomplicated *P. knowlesi* should be treated with chloroquine or an ACT drug and complicated *P. knowlesi* with IVA.

#### Interactions

The effect of food on the absorption and pharmacokinetics of anti-malarials is important (Table 3). In general, orally administered agents are given with food to enhance absorption. The exception is dihydroartemisinin/piperaquine which should be administered fasting, as studies have shown that this reduces the possible QTc prolongation impact of piperaquine [Eurartesim – EMA summary of Product Characteristics]. Conversely, for artemether/lumefantrine a fatty meal is recommended to achieve therapeutic levels of the fat soluble lumefantrine.

The most prominent drug/drug interaction is the effect on QTc-prolongation with quinine, artemether/lumefantrine as well as with mefloquine. The effect of the individual drugs can be enhanced if they are used together, consecutively, or at the same time as other cardioactive drugs, such as ketoconazole or phenothiazines. Quinine and atovaquone/proguanil can enhance the effect of anticoagulants. Concomitant treatment with rifampicin, metoclopramide or tetracycline can result in a lower plasma concentration of atovaquone/proguanil.

Treatment of HIV/malaria co-infected patients has potential for drug interactions (Table 7). A recent paper examining the co-administration of artemether/lumefantrine with lopinavir/ritonavir, showed significant increases in lumefantrine blood levels but decreases in artemether blood levels [125]. Further studies are needed to assess the clinical importance of antiretroviral antimalarial drug interactions.

**Table 7:** Drug interactions between antimalarial and antiretroviral medication

**Drugs**	**Mefloquine**	**Atovaquone**	**Proguanil**	**Chloroquine**	**Artemisinins**	**Quinine**
**Protease inhibitors**						
Saquinavir	**-**	**+**	**-**	**-**	**?**	**+**
Ritonavir	**-**	**+**	**+**	**+**	**+**	**+**
Indinavir	**-**	**+**	**-**	**-**	**?**	**+**
Nelfinavir	**-**	**+**	**-**	**-**	**?**	**+**
Amprenavir	**-**	**+**	**-**	**-**	**?**	**+**
Lopinavir	**-**	**+**	**-**	**-**	**+**	**+**
Atazanavir	**-**	**+**	**-**	**-**	**?**	**+**
**NNRTIs**^(1)^						
Nevirapine, efavirenz and others	**-**	**-**	**-**	**-**	**?**	**+**
**NRTIs**^(2)^						
Zidovudine and others	**-**	**-**	**-**	**-**	**-**	**-**

(1) Non-Nucleoside Reverse Transcriptase Inhibitors.

(2) Nucleoside Reverse Transcriptase Inhibitors.

**-**No clinically significant interaction, or interaction unlikely based on knowledge of drug metabolism.

**+**Potential interaction that may require dose monitoring, alteration of drug dosage or timing of administration.

## Conclusions

All hospitals in Europe should have plans for diagnosing and managing patients with malaria. Obtaining a travel history is mandatory for all patients with fever. If RDTs indicate the diagnosis of malaria but microscopy cannot be performed adequate treatment should be started straight away and the patient promptly transferred to a health care facility where microscopy can be done. In the case of a negative RDT but high suspicion of malaria, patients should also be transferred to a centre with expertise in the microscopic diagnosis of malaria. ACT should be available for treatment of uncomplicated malaria and intravenous artesunate available for severe or complicated malaria. Intravenous quinine should be available if it is not possible to maintain a stock of intravenous artesunate.

A standing committee is proposed to harmonize guidelines as far as possible, whilst recognizing the need in some areas for individual/country-specific decision-making. The main recommendations for non-specialist physicians are summarized on Table 8.

**Table 8:** Recommendations


**When should malaria be suspected?**	In all ill patients with a travel history of visiting amalaria endemic area in the last year, especially inthe last 3 months.
**Initial diagnosis**	Microscopy of thick and thin Giemsa stained bloodfilms. Use of a rapid diagnostic test can be used ifmicroscopy is unavailable initially, but follow upby microscopy is essential.
**Monitoring after diagnosis**	Microscopy of thick and thin Giemsa stained bloodfilms. If this skill is not available the patient shouldbe transferred to a level where microscopy can be performed.
**Treatment of uncomplicated malaria**	See tables 1 and 2. Artemisinin combination therapy or atovaquone-proguanil are first linetreatment options for *P.falciparum* and can also be used for the other species. Chloroquine is the first line treatment for other malaria species.
**Treatment of complicated malaria**	Artesunate or quinine intravenously. If available,artesunate is preferable to quinine. These drugs must be available at the health care facility managing patients with malaria.
**Treatment of non-falciparum malaria**	Chloroquine is the drug of choice and ACT a pragmatic alternative. In case of chloroquine resistance an ACT is second line treatment.
	Primaquine is given after testing for G6PD at adose of 30 mg per day for patients infected in Southeast Asia; otherwise 15 mg/day.
**Managing uncomplicated malaria**	Patients with *P. falciparum* malaria should preferably be managed as in-patients. Under certain circumstances out-patient management may be acceptable provided there are daily assessments and daily blood films for parasitaemia until clinical and parasitological cure.
**Managing complicated malaria**	Patients with complicated *P. falciparum* malaria (Table 4) should be managed as inpatients in anIntensive Care Unit.
	Patients receiving intravenous artesunate should be monitored twice weekly for 4 weeks following IVA for hemolysis and leucopenia.

## Competing interests

Gerd Burchard has received speakers' honoraria from GlaxoSmithKline, Novartis Pharma and Sigma-Tau. Peter L. Chiodini has received speakers' honoraria from GlaxoSmithKline. Patricia Schlagenhauf has received speakers' honoraria and research funds from GlaxoSmithKline, speakers honoraria,research funds and consultancy fees from F. Hoffmann-La Roche and she is a member of the sigma-tau advisory board. Eskild Petersen has received speakers a honoraria from GlaxoSmithKline.

## Authors' contributions

EP coordinated the project and manuscript, all authors contributed sections to the manuscript and all authors read and approved the final manuscript.
